# Supplementary material for: Towards personalised anti-microbial and immune approaches to infections in acute care. Can real-time genomic-informed diagnosis of pathogens, and immune-focused therapies improve outcomes for patients? An observational, experimental study protocol
Source: PLoS One. 2024 Mar 29;19(3):e0298425. doi: 10.1371/journal.pone.0298425 (PMC10980213; doi:10.1371/journal.pone.0298425)
Supplement: Attachment — Submitted filename: plos one.docx [file pone.0298425.s001.docx]

I recommend the study protocol for acceptance but the need the minor changes related to the study

Mention the proper heading for the following such as

# Builds on previous work

# Differs from previous work
