# Supplementary material for: Towards personalised anti-microbial and immune approaches to infections in acute care. Can real-time genomic-informed diagnosis of pathogens, and immune-focused therapies improve outcomes for patients? An observational, experimental study protocol
Source: PLoS One. 2024 Mar 29;19(3):e0298425. doi: 10.1371/journal.pone.0298425 (PMC10980213; doi:10.1371/journal.pone.0298425)
Supplement: Attachment — Submitted filename: Response to Reviewers.docx [file pone.0298425.s002.docx]

Dear Dr Liu,

Many thanks for your consideration of our manuscript and helpful comments for which we are very grateful. Please see the table below for our responses to the points raised.

| **Academic editor/reviewer’s comment** | **Response** |
| --- | --- |
| 1. The authors state "there is currently significant delay between sample collection and report read out, with average turnaround times for results being three days (13), due to the time taken to complete standard microbial culture and sensitivity assays." It is inappropriate to over generalize that turnaround time are 3 days as some assays provide quick turnaround. Also to make such statement the authors should define the clinical settings, e.g., inpatient and outpatient. The author's statement may be misleading. In addition, the turnaround time and delay in reporting is due to multiple factors, including but not limited to the medical systems and their efficiencies, clinical team, and labs. The authors statement should be modified to give the audience a whole picture. | We recognise the point addressed here and have expanded on and clarified this within the revised text.  The relevant section now reads:  “However, current techniques often contribute to significant delay between sample collection and report read out, termed laboratory turnaround time (LTAT), with an average LTAT of three days being reported for culture-based approaches in an inpatient population (13). While some of this delay will be due to common factors such as the efficiency of clinical teams and hospital systems, the time taken for traditional microbial culture is also a significant component. Other assays such as antigen-based testing can provide quicker results but require selective testing and are not available for all pathogens.  Modern genomic and molecular-based methods have the potential…” |
| 2. The authors state "Modern genomic and molecular-based methods have the potential to rapidly identify not only the exact type of pathogen, but also genetic mutations which might affect virulence and antibiotic resistance (14)." The authors are suggested to add pros and cons of molecular testings. Besides, more references should be added. For instance, the following references may be relevant, it is optional to cite them. They can support the advantages of molecular assays and facilitate discussion on pros and cons of these tests comparing with other traditional tests.  Laboratory diagnosis of CNS infections in children due to emerging and re-emerging neurotropic viruses. Pediatr Res. 2023 Dec 2. doi: 10.1038/s41390-023-02930-6. Epub ahead of print. PMID: 38042947.  Novel HBV recombinants between genotypes B and C in 3'-terminal reverse transcriptase (RT) sequences are associated with enhanced viral DNA load, higher RT point mutation rates and place of birth among Chinese patients. Infect Genet Evol. 2018 Jan;57:26-35. doi: 10.1016/j.meegid.2017.10.023. Epub 2017 Oct 27. PMID: 29111272. | We agree that readers would benefit from a fuller exploration of the pros and cons of molecular testing. The revised text is as follows:  “Modern genomic and molecular-based methods have the potential to rapidly identify not only the exact type of pathogen, but also genetic mutations which might affect virulence (14) and antibiotic resistance (15, 16). The pathogen-agnostic nature of metagenomic next-generation sequencing (mNGS) can also allow for the detection of rare or emerging pathogens (17, 18). However, how best to interpret the clinical significance of positive results is uncertain given organisms identified may represent colonisation rather than infection (16). It is also currently unclear whether these cutting-edge techniques can be applied across all common infections in hospitals or community settings and be embedded into a clinical service.”  We have included the references suggested by the reviewer. |
| 3. Mention the proper heading for the following such as:  Builds on previous work  Differs from previous work | We have added this in as a summary section after the introduction.  Builds on previous work:   - Causative pathogens are currently not identified in over half of patients hospitalised with infection - Up to 50% of antibiotic prescriptions are estimated to be inappropriate - Metagenomic next generation sequencing techniques can identify organisms with high sensitivity - Severe infection is known to cause prolonged immune dysfunction in survivors   Differs from previous work:   - Combination of microbiological and clinical data together with immune cell function in one study, allowing for analysis into the interplay of these factors. - Broad scope investigating all acute infection. This will allow for differences depending on infection source to be explored. |
| **Journal Requirements** |  |
| 1. Please ensure that your manuscript meets PLOS ONE's style requirements, including those for file naming. | Edited style and formatting as per the included templates, many thanks. |
| 2. Thank you for stating the following financial disclosure:  "This study is funded by the National Institute for Health and Care Research (NIHR) Birmingham Biomedical Research Centre (BRC). The views expressed are those of the author(s) and not necessarily those of the NIHR or the Department of Health and Social Care.  It is also supported by PIONEER, the Health Data Research UK (HDR-UK) Health Data Research Hub in acute care. HDR-UK is an initiative funded by the UK Research and Innovation, Department of Health and Social Care (England) and the devolved administrations, and leading medical research charities."    Please state what role the funders took in the study. If the funders had no role, please state: ""The funders had no role in study design, data collection and analysis, decision to publish, or preparation of the manuscript.""  If this statement is not correct you must amend it as needed. | Financial disclosure updated to include this as below: “This study is funded by the National Institute for Health and Care Research (NIHR) Birmingham Biomedical Research Centre (BRC). The views expressed are those of the author(s) and not necessarily those of the NIHR or the Department of Health and Social Care.  It is also supported by PIONEER, the Health Data Research UK (HDR-UK) Health Data Research Hub in acute care. HDR-UK is an initiative funded by the UK Research and Innovation, Department of Health and Social Care (England) and the devolved administrations, and leading medical research charities. The funders had no role in study design, data collection and analysis, decision to publish, or preparation of the manuscript.” |
| 3. When completing the data availability statement of the submission form, you indicated that you will make your data available on acceptance. We strongly recommend all authors decide on a data sharing plan before acceptance, as the process can be lengthy and hold up publication timelines. Please note that, though access restrictions are acceptable now, your entire data will need to be made freely accessible if your manuscript is accepted for publication. This policy applies to all data except where public deposition would breach compliance with the protocol approved by your research ethics board. If you are unable to adhere to our open data policy, please kindly revise your statement to explain your reasoning and we will seek the editor's input on an exemption. Please be assured that, once you have provided your new statement, the assessment of your exemption will not hold up the peer review process. | This is a protocol paper and as such no data is available with this protocol.  When we publish the results of the study, we will include a data sharing plan and statement, and these will be in keeping with the journals requirements. |

We have also updated our conflict-of-interest statement as follows, and have updated this within the submission platform:
Elizabeth Sapey needs to declare funding from HDR-UK, The Wellcome Trust, MRC, NIHR, and Asthma + Lung UK. These fundings are not commercial but charitable and do not relate to the study but to Elizabeth Sapey. Alan McNally reports funding from UK Research and Innovation and Wellcome Trust. No other authors have interests to declare.

We hope that the above changes address the revisions suggested, however please let us know if you have any further comments, and thank you again for your time and input in consideration of our manuscript.

Kind regards,

Samuel Quarton

**References**

13. Tabak Ying P, Vankeepuram L, Ye G, Jeffers K, Gupta V, Murray Patrick R. Blood Culture Turnaround Time in U.S. Acute Care Hospitals and Implications for Laboratory Process Optimization. J Clin Microbiol. 2018;56(12):e00500-18

14. Liu B, Yang JX, Yan L, Zhuang H, Li T. Novel HBV recombinants between genotypes B and C in 3'-terminal reverse transcriptase (RT) sequences are associated with enhanced viral DNA load, higher RT point mutation rates and place of birth among Chinese patients. Infect Genet Evol. 2018 Jan;57:26-35. doi: 10.1016/j.meegid.2017.10.023. Epub 2017 Oct 27. PMID: 29111272.

15. Geurtsen J, de Been M, Weerdenburg E, Zomer A, McNally A, Poolman J. Genomics and pathotypes of the many faces of Escherichia coli. FEMS Microbiology Reviews. 2022;46(6):fuac031.

16. Chiu CY, Miller SA. Clinical metagenomics. Nat Rev Genet. 2019;20(6):341-55.

17. Laboratory diagnosis of CNS infections in children due to emerging and re-emerging neurotropic viruses. Pediatr Res. 2023 Dec 2. doi: 10.1038/s41390-023-02930-6. Epub ahead of print. PMID: 38042947

18. Shi Y, Chen J, Shi X, Hu J, Li H, Li X, Wang Y, Wu B. A case of chlamydia psittaci caused severe pneumonia and meningitis diagnosed by metagenome next-generation sequencing and clinical analysis: a case report and literature review. BMC Infect Dis. 2021 Jun 30;21(1):621. doi: 10.1186/s12879-021-06205-5. PMID: 34193063; PMCID: PMC8243071
